# Adaptive neural network prescribed performance control for dual switching nonlinear time-delay system

**DOI:** 10.1038/s41598-023-35307-0

**Published:** 2023-05-19

**Authors:** Qianqian Mu, Fei Long, Bin Li

**Affiliations:** 1 College of Big Data and Information Engineering, Guizhou University, Guiyang, 550025 Guizhou China; 2 School of Mathematics and Big Data, Guizhou Education University, Guiyang, 550018 Guizhou China; 3 School of Artificial Intelligence and Electrical Engineering, Guizhou Institute of Technology, Guiyang, 550003 Guizhou China; 4 Guizhou Key Laboratory of Artificial Intelligence and Intelligent Control, Guiyang, 550003 Guizhou China; 5 China Tower Corporation Limited Guizhou Provincial Branch, Guiyang, 550003 Guizhou China

**Keywords:** Electrical and electronic engineering, Applied mathematics, Computer science

## Abstract

This paper investigates the adaptive neural network prescribed performance control problem for a class of dual switching nonlinear systems with time-delay. By using the approximation of neural networks (NNs), an adaptive controller is designed to achieve tracking performance. Another research point of this paper is tracking performance constraints which can solve the performance degradation in practical systems. Therefore, an adaptive NNs output feedback tracking scheme is studied by combining the prescribed performance control (PPC) and backstepping method. With the designed controller and the switching rule, all signals of the closed-loop system are bounded, and the tracking performance satisfies the prescribed performance.

## Introduction

Dual switching systems are a class of hybrid systems that have both deterministic switching subsystems and stochastic switching subsystems. Its switching mechanism is more complex than the traditional switching system, and contains both deterministic and random switching signals. Since Markov stochastic processes are often used to describe its stochastic subsystems, it is also known as switching Markov jump system^1,2^. It has been gradually applied in recent years to model systems such as network control systems^3,4^, battery energy storage system^5^, fault tolerant systems^6^, etc.

The control problem for dual switching nonlinear system has received a lot of attention. Bolzern et al.^1^ and Song et al.^2^ studied the almost sure stability of Markov jump linear systems. Reference^7^ provided sufficient conditions in terms of matrix inequalities for mean square stability and mean square stabilizability of discrete-time dual switching systems. The almost sure stability for discrete dual switching system and continuous dual switching system is studied respectively in Refs.^8–10^.

As the switching mechanisms for practical applications are becoming more and more complex and the requirements for the system are higher, the transient performance of the system has attracted widespread attention. To achieve the prescribed performance, the studies for switched systems mainly used neural network adaptive control^11,12^, dynamic surface control^8,13^, fuzzy control^14–17^ and other schemes^18^. Time-delay, as an important factor affecting system performance, is also widespread in practical systems. Therefore, research for time-delay systems is also meaningful. The research results for time-delay systems are numerous. In order to investigate the stability of switched time-delay systems, the Lyapunov–Krasovskii functions^19,20^, average dwell time (ADT) method^21^, neural network-based adaptive control^22,23^.

**Figure 1:**
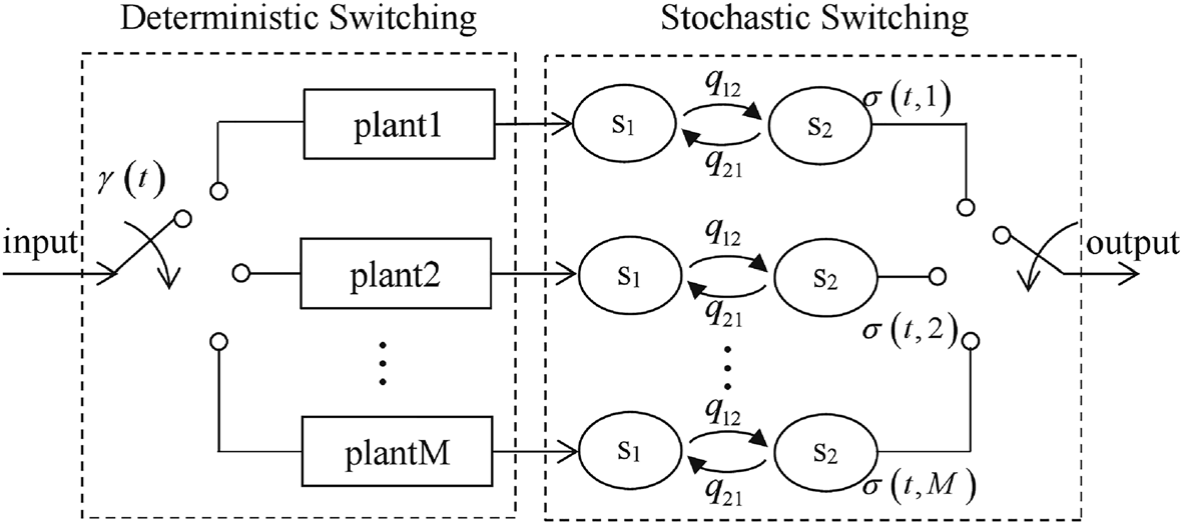
a dual switching system model of network control system.

Figure 1 shows a dual switching system model applied to the scheduling of a network control system with packet dropout. Assume that there are M plants that are controlled by a regulator. The scheduling signal $\gamma \left( t \right)$ takes values in the set M and only one plant is scheduled at a time. The data transmission over the network is affected by a stochastic fault that is modeled by a Markov process $\sigma \left( t \right)$ taking values in the set $N = \left\{ {1,2} \right\}$. Let $\sigma \left( t \right) = 1$ represent the fault-free mode when all packets are transmitted correctly, and $\sigma \left( t \right) = 2$ represent the packet dropout mode when no packets are sent. In this dual switching model^3^, the scheduling signal $\gamma \left( t \right)$ needs to be designed to satisfy the stability of the data transmission in the network.

Motivated by the above observations, the following questions have drawn our attention: (1) How to design an adaptive controller when both unmeasurable states and time-delay exist in a dual-switching nonlinear system? (2) How to achieve stabilization when both transient stability and system stability are required? Those questions will be solved in this paper. In this paper, adaptive neural network prescribed performance control for a class of dual switching nonlinear systems with time-delay is investigated. The backstepping method, multiple Lyapunov functions, and prescribed performance theory are applied to design an adaptive neural controller. Then we prove that all signals in the closed-loop system are bounded and the steady-state and transient performance are satisfied under the joint action of the designed switching rule and the controller. The main contributions of this paper are as follows.

1. Compared with previous works^22,23^, this paper studies a class of dual-switching nonlinear systems with more complex switching mechanisms and with both time-delay and unobservable states. The system model studied is more general and challenging than the present results. The approximation capability of neural networks is used to design adaptive controllers to stabilize the system.
2. Previous studies^9,10^ focused on the stability of dual-switching systems. However, these works could not guarantee the predefined transient and steady-state performance, and none of them considered the time-delay. The control scheme proposed in this paper successfully solves the PPC problem for dual switching nonlinear systems with time-delay.
3. Adaptive laws for each subsystems were designed to reduce the conservatism introduced^14,18^ by the common adaptive law for all subsystems.

The rest of the paper is organized as follows. The problem formulation and the preliminaries are presented in “Problem formulation and preliminaries” section. The main results are derived in in “Controller design” and “Stability analysis” sections. An example is shown in “Numerical simulation” section. “Conclusion” section concludes this paper.

## Problem formulation and preliminaries

### Problem formulation

Consider the following dual switching nonlinear system with time-delay.

1 $$\begin{aligned} \left\{ \begin{array}{l} {{\dot{x}}_i} = f_{i,\sigma \left( t \right) }^{\gamma \left( t \right) }\left( {{{\bar{x}}_i}} \right) + g_{i,\sigma \left( t \right) }^{\gamma \left( t \right) }\left( {{{\bar{x}}_i}} \right) {x_{i + 1}} + h_{i,\sigma \left( t \right) }^{\gamma \left( t \right) }\left( {{{\bar{x}}_i}\left( {{\tau _i}} \right) } \right) ,\;1 \le i \le n - 1,\\ {{\dot{x}}_n} = f_{n,\sigma \left( t \right) }^{\gamma \left( t \right) }\left( x \right) + g_{n,\sigma \left( t \right) }^{\gamma \left( t \right) }\left( x \right) u_{\sigma \left( t \right) }^{\gamma \left( t \right) } + h_{n,\sigma \left( t \right) }^{\gamma \left( t \right) }\left( {x\left( \tau \right) } \right) ,\\ y = {x_1}, \end{array} \right. \end{aligned}$$

where ${\bar{x}_i} = {\left( {{x_1}, \ldots ,{x_i}} \right) ^T}$ and $x = {\left( {{x_1}, \ldots ,{x_n}} \right) ^T} \in {R^n}$ are the system state variables. ${\bar{x}_i}\left( {{\tau _i}} \right) = {\left( {{x_1}\left( {t - {\tau _1}} \right) , \ldots ,{x_i}\left( {t - {\tau _i}} \right) } \right) ^T}$ and $x\left( \tau \right) = \left( {{x_1}\left( {t - {\tau _1}} \right) , \ldots ,{x_n}\left( {t - {\tau _n}} \right) } \right) \in {R^n}$ are the time-delay of the system states. ${\tau _i}$ stands for a stochastic delay and has the upper bounds ${\tau _m}$. The nonlinear terms $f_{\sigma \left( t \right) }^{\gamma \left( t \right) }$, $g_{\sigma \left( t \right) }^{\gamma \left( t \right) }$ and the nonlinear time-delay terms $h_{\sigma \left( t \right) }^{\gamma \left( t \right) }$ are continuous and unknown. $u_{\sigma \left( t \right) }^{\gamma \left( t \right) }$ is the control input. Denote for simplicity, $f_{\sigma \left( t \right) }^{\gamma \left( t \right) } = f_\sigma ^\gamma$, $h_{\sigma \left( t \right) }^{\gamma \left( t \right) } = h_\sigma ^\gamma$, $g_{\sigma \left( t \right) }^{\gamma \left( t \right) } = g_\sigma ^\gamma$ and $u_{\sigma \left( t \right) }^{\gamma \left( t \right) } = u_\sigma ^\gamma$. ${y_d}$ is a desired reference signal.

#### Assumption 1

The reference signal ${y_d}$ is smooth and ${y_d}$, ${\dot{y}_d}$ and ${\ddot{y}_d}$ are bounded. There exists a positive constant ${Y_D}$ such that $\left( {{y_d},{{\dot{y}}_d},{{\ddot{y}}_d}} \right)$ belongs to the compact ${\Omega _y}$, where ${\Omega _y} = \left\{ {\left( {{y_d},{{\dot{y}}_d},{{\ddot{y}}_d}} \right) :y_d^2 + \dot{y}_d^2 + \ddot{y}_d^2 \le {Y_D}} \right\}$.

#### Assumption 2

For $i = 1, \ldots ,n$, the nonlinear function $h_{i,\sigma \left( t \right) }^{\gamma \left( t \right) }\left( {{{\bar{x}}_i}\left( {{\tau _i}} \right) } \right)$ satisfies the following inequality:

$$\left| {h_{i,\sigma \left( t \right) }^{\gamma \left( t \right) }\left( {{{\bar{x}}_i}\left( {t - {\tau _i}} \right) } \right) } \right| \le \left| {{h_i}\left( {{{\bar{x}}_i}\left( {t - {\tau _i}} \right) } \right) } \right| \le \sum \nolimits _{j = 1}^i {{\beta _{i,j}}\left( {{x_j}\left( {t - {\tau _j}} \right) } \right) } .$$

#### Remark 1

Assumption 1 is common in signal tracking with specified performance^23,24^. Assumption 2 is a general assumption in nonlinear time-delay systems^25,26^. In fact, for any continuous function $h\left( {{x_1}, \ldots ,{x_n}} \right) :{R^n} \rightarrow R$, there exist positive smooth functions ${h_1}\left( {{x_1}} \right) , \ldots ,{h_n}\left( {{x_n}} \right)$ such that $h\left( {{x_1}, \ldots ,{x_n}} \right) \le \sum \nolimits _{i = 1}^n {{h_i}\left( {{x_i}} \right) }$^27^.

#### Assumption 3

For $i = 1, \ldots ,n$, there exits ${\omega _j}$ such that $\sum \nolimits _{j = 1}^N {{\omega _j}} = 1,{\omega _j} \in \left( {0,1} \right)$, satisfying $0 < {g_i} \le \left| {{g_i}\left( {{{\bar{x}}_i}} \right) } \right| = \left| {\sum \nolimits _{j = 1}^N {{\omega _j}{g_{i,j}}\left( {{{\bar{x}}_i}} \right) } } \right|$, with ${g_i}$ being constants and ${g_i}\left( {{{\bar{x}}_i}} \right) > 0$.

### Prescribed performance control

To achieve the prescribed transient and steady state behavioral bounds on the tracking errors ${s_1}\left( t \right) = y\left( t \right) - {y_d}\left( t \right)$, guaranteeing the objective is equivalent to

2 $$\begin{aligned} - \underline{\delta } \rho \left( t \right)< {s_1}\left( t \right) < \overline{\delta } \rho \left( t \right), \end{aligned}$$

for all $t \ge 0$, where $\rho \left( t \right) = \left( {{\rho _0} - {\rho _\infty }} \right) {e^{ - \beta t}} + {\rho _\infty }$, the constants satisfy $\underline{\delta },\overline{\delta } ,\beta > 0$ and $0< {s_1}\left( 0 \right) < \rho \left( 0 \right)$. Choose a smooth strictly increasing function $\nu \left( t \right) = {S^{ - 1}}\left( {\frac{{{s_1}}}{\rho }} \right) = \frac{1}{2}\ln \left( {\frac{{{s_1}}}{\rho } + \underline{\delta }} \right) - \frac{1}{2}\ln \left( {\overline{ \delta } - \frac{{{s_1}}}{\rho }} \right)$ as the conversion function. Then, we have $\dot{\nu }= r\left( {{{\dot{s}}_1} - \frac{{\dot{\rho }{s_1}}}{\rho }} \right)$, where $r = \frac{1}{2}\left( {\frac{1}{{{s_1} + \underline{\delta } \rho }} - \frac{1}{{{s_1} - \overline{\delta } \rho }}} \right)$. It can be claimed that prescribed performance shown in (2) is guaranteed if the $\nu \left( t \right)$ is bounded.

### Radial basis function neural network

Let *F*(*z*) be an unknown continuous function defined on a given compact set ${\Omega _z}$. The RBFNN can be used to approximate *F*(*z*) on the compact set ${\Omega _z}$. It can be expressed as

$$\begin{aligned} F(z) = {\theta ^T}\varphi (z) + \xi (z), \end{aligned}$$

where $\xi (z)$ is the approximate error, which is bounded. $$\theta = \left[ {\begin{array}{*{20}{c}} {{\theta _1}}&{{\theta _2}}&\cdots&{{\theta _l}} \end{array}} \right] ,l > 1$$ denotes weight vector, and $$\varphi (z) = {\left[ {\begin{array}{*{20}{c}} {{\varphi _1}(z)}&{{\varphi _2}(z)}&\cdots&{{\varphi _l}(z)} \end{array}} \right] ^T}$$ is the basis function vector with ${\varphi _i}(z)$ being the Gaussian function in the form

$$\begin{aligned} {\varphi _i}(z) = \exp \left( {\frac{{ - {{\left\| {z - {c_i}} \right\| }^2}}}{{2\sigma _i^2}}} \right) ,i = 1,2, \ldots ,l, \end{aligned}$$

where ${c_i}$ is the center of the radial basic function and ${\sigma _i}$ is the width of the Gaussian function.

## Controller design

To begin with the controller design procedure, let us introduce the following coordinates transformation:

3 $$\begin{aligned} \left\{ \begin{array}{l} {e_1} = \nu ,\\ {e_i} = {x_i} - {z_i},\\ {\chi _i} = {z_i} - {\alpha _{i - 1}},\;i = 2,3, \ldots ,n, \end{array} \right. \end{aligned}$$

where ${e_i}$ is the error, ${z_i}$ is the output state that is obtained through first-order filter, ${\alpha _{i - 1}}$ is the virtual controller. The first-order filters are given as ${\varsigma _i}{\dot{z}_i} + {z_i} = {\alpha _{i - 1}},{z_i}\left( 0 \right) = {\alpha _{i - 1}}\left( 0 \right) ,i = 2,3, \ldots ,n$, where ${\varsigma _i}$ is a positive design parameter.

*Step 1* For $i = 1$, ${\dot{e}_1}$ is directly obtained from (1) and (3), ${{\dot{e}}_1} = r\left( {f_{1,\sigma }^\gamma \left( {{{\bar{x}}_1}} \right) + g_{1,\sigma }^\gamma \left( {{{\bar{x}}_1}} \right) {x_2} + h_{1,\sigma }^\gamma \left( {{{\bar{x}}_1}\left( {{\tau _1}} \right) } \right) - {{\dot{y}}_d} - {{\dot{\rho }{s_1}} \big /\rho }} \right)$. Consider the following Lyapunov function

$$\begin{aligned} {V_1} = \frac{1}{2}e_1^2 + {l_1} + \frac{{\tilde{\theta }_1^T{{\tilde{\theta }}_1}}}{{2{\mu _1}}}, \end{aligned}$$

where $\tilde{\theta }= \theta - \hat{\theta }$ and ${l_1} = \frac{1}{2}{e^{ - {k_1}\left( {t - {\tau _1}} \right) }}\int _{t - {\tau _1}}^t {{e^{{k_1}s}}\beta _{1,1}^2\left( {{x_1}\left( s \right) } \right) ds}$. Taking the derivative of ${V_1}$, denoted as ${\dot{V}_1}$,

$$\begin{aligned} {{\dot{V}}_1} = {e_1}{{\dot{e}}_1} - {k_1}{l_1} + \frac{1}{2}{e^{{k_1}{\tau _1}}}\beta _{1,1}^2\left( {{x_1}\left( t \right) } \right) - \frac{1}{2}\beta _{1,1}^2\left( {{x_1}\left( {t - {\tau _1}} \right) } \right) - \frac{{\tilde{\theta }_1^T{{\dot{ \hat{\theta }}}_1}}}{{{\mu _1}}}. \end{aligned}$$

For $t \in \left[ {{t_k},{t_{k + 1}}} \right)$, the *k*-th determined subsystem is activated when $\gamma \left( t \right) = k$, denoted by ${\gamma _k}$.

4 $$\begin{aligned} \begin{aligned} \dot{V}_{1,\sigma }^{{\gamma _k}}&= {e_1}r\left( {f_{1,\sigma }^{{\gamma _k}}\left( {{{\bar{x}}_1}} \right) + g_{1,\sigma }^{{\gamma _k}}\left( {{{\bar{x}}_1}} \right) {x_2} + h_{1,\sigma }^{{\gamma _k}}\left( {{{\bar{x}}_1}\left( {{\tau _1}} \right) } \right) - {{\dot{y}}_d} - \frac{{\dot{\rho }{s_1}}}{\rho } + \frac{1}{{2{e_1}r}}{e^{{k_1}{\tau _1}}}\beta _{1,1}^2\left( {{x_1}} \right) } \right) \\&\quad - {k_1}{l_1} - \frac{1}{2}\beta _{1,1}^2\left( {{x_1}\left( {t - {\tau _1}} \right) } \right) - \frac{{{{\left( {\tilde{\theta }_{1,\sigma }^{{\gamma _k}}} \right) }^T}\dot{\hat{\theta }} _{1,\sigma }^{{\gamma _k}}}}{{\mu _{1,\sigma }^{{\gamma _k}}}}. \end{aligned} \end{aligned}$$

From Assumption 2, we can get that

5 $$\begin{aligned} {e_1}rh_{1,\sigma }^{{\gamma _k}}\left( {{{\bar{x}}_1}\left( {{\tau _1}} \right) } \right) \le \frac{1}{2}e_1^2{r^2} + \frac{1}{2}\beta _{1,1}^2\left( {{x_1}\left( {t - {\tau _1}} \right) } \right) . \end{aligned}$$

According to (3), ${x_2} = {e_2} + {\chi _2} + {\alpha _1}$. Then, it holds

6 $$\begin{aligned} {e_1}rg_{1,\sigma }^{{\gamma _k}}\left( {{x_1}} \right) \left( {{e_2} + {\chi _2} + {\alpha _1}} \right) \le e_1^2{r^2}{\left( {g_{1,\sigma }^{{\gamma _k}}\left( {{x_1}} \right) } \right) ^2} + \frac{1}{2}e_2^2 + \frac{1}{2}\chi _2^2 + {e_1}rg_{1,\sigma }^{{\gamma _k}}\left( {{x_1}} \right) {\alpha _1}. \end{aligned}$$

Substitute (5) and (6) into (4),

7 $$\begin{aligned} \dot{V}_{1,\sigma }^{{\gamma _k}} \le {e_1}rF_{1,\sigma }^{{\gamma _k}}\left( {{{\bar{X}}_1}} \right) + {e_1}rg_{1,\sigma }^{{\gamma _k}}\left( {{x_1}} \right) {\alpha _1} + \frac{1}{2}e_1^2{r^2} + \frac{1}{2}e_2^2 + \frac{1}{2}\chi _2^2 - {k_1}{l_1} - \frac{{{{\left( {\tilde{\theta }_{1,\sigma }^{{\gamma _k}}} \right) }^T}\dot{\hat{\theta }} _{1,\sigma }^{{\gamma _k}}}}{{\mu _{1,\sigma }^{{\gamma _k}}}}. \end{aligned}$$

Denote $F_{1,\sigma }^{{\gamma _k}}\left( {{{\bar{X}}_1}} \right) = f_{1,\sigma }^{{\gamma _k}}\left( {{x_1}} \right) + {e_1}r{\left( {g_{1,\sigma }^{{\gamma _k}}\left( {{x_1}} \right) } \right) ^2} - {\dot{y}_d} - {{\dot{\rho }{s_1}} \big / \rho } + \frac{1}{{2{e_1}r}}{e^{{k_1}{\tau _1}}}\beta _{1,1}^2\left( {{x_1}} \right)$, where ${\bar{X}_1} = \left( {{x_1},{y_d},{{\dot{y}}_d},\rho ,\dot{\rho }} \right)$. We approximate the unknown functions $F_{1,\sigma }^{{\gamma _k}}\left( {{{\bar{X}}_1}} \right)$ by using the RBF neural networks. $F_{1,\sigma }^{{\gamma _k}}\left( {{{\bar{X}}_1}} \right) = {\left( {\theta _{1,\sigma }^{{\gamma _k}}} \right) ^T}\varphi _{1,\sigma }^{{\gamma _k}}\left( {{{\bar{X}}_1}} \right) + \xi _{1,\sigma }^{{\gamma _k}}\left( {{{\bar{X}}_1}} \right)$, where the approximation error $\xi _{1,\sigma }^{{\gamma _k}}\left( {{{\bar{X}}_1}} \right)$ is bounded and $\xi _{1,\sigma }^{{\gamma _k}}\left( {{{\bar{X}}_1}} \right) \le {\xi _1}$. Using the Young’s Inequality, one has

8 $$\begin{aligned} \begin{aligned} {e_1}rF_{1,\sigma }^{{\gamma _k}}\left( {{{\bar{X}}_1}} \right)&= {e_1}r\left( {{{\left( {\theta _{1,\sigma }^{{\gamma _k}}} \right) }^T}\varphi _{1,\sigma }^{{\gamma _k}}\left( {{{\bar{X}}_1}} \right) + \xi _{1,\sigma }^{{\gamma _k}}\left( {{{\bar{X}}_1}} \right) } \right) \\&= {e_1}r{\left( {\theta _{1,\sigma }^{{\gamma _k}}} \right) ^T}\varphi _{1,\sigma }^{{\gamma _k}}\left( {{{\bar{X}}_1}} \right) + {e_1}r\xi _{1,\sigma }^{{\gamma _k}}\left( {{{\bar{X}}_1}} \right) \\&\le {e_1}r{\left( {\theta _{1,\sigma }^{{\gamma _k}}} \right) ^T}\varphi _{1,\sigma }^{{\gamma _k}}\left( {{{\bar{X}}_1}} \right) + \frac{1}{2}e_1^2{r^2} + \frac{1}{2}\xi _1^2. \end{aligned} \end{aligned}$$

By (7) and (8), we have

$$\begin{aligned} \dot{V}_{1,\sigma }^{{\gamma _k}} \le {e_1}r{\left( {\theta _{1,\sigma }^{{\gamma _k}}} \right) ^T}\varphi _{1,\sigma }^{{\gamma _k}}\left( {{{\bar{X}}_1}} \right) + {e_1}rg_{1,\sigma }^{{\gamma _k}}\left( {{x_1}} \right) {\alpha _1} + e_1^2{r^2} + \frac{1}{2}{\left( {\xi _{1,\sigma }^{{\gamma _k}}} \right) ^2} + \frac{1}{2}e_2^2 + \frac{1}{2}\chi _2^2 - {k_1}{l_1} - \frac{{{{\left( {\tilde{\theta }_{1,\sigma }^{{\gamma _k}}} \right) }^T}\dot{\hat{\theta }} _{1,\sigma }^{{\gamma _k}}}}{{\mu _{1,\sigma }^{{\gamma _k}}}}. \end{aligned}$$

For each stochastic subsystem, design the adaptive law and the virtual control as

9 $$\begin{aligned}{} & {} \dot{\hat{\theta }}_{1,\sigma }^{{\gamma _k}} = \mu _{1,\sigma }^{{\gamma _k}}{e_1}r\varphi _{1,\sigma }^{{\gamma _k}}\left( {{{\bar{X}}_1}} \right) , \end{aligned}$$

10 $$\begin{aligned}{} & {} {\alpha _1} = \frac{{ - {\lambda _1}{e_1} - {e_1}{r^2} - r{{\left( {\hat{\theta }_{1,\sigma }^{{\gamma _k}}} \right) }^T}\varphi _{1,\sigma }^{{\gamma _k}}\left( {{{\bar{X}}_1}} \right) }}{{rg_{1,\sigma }^{{\gamma _k}}\left( {{x_1}} \right) }}. \end{aligned}$$

With the adaptive law (9) and (10), $\dot{V}_{1,\sigma }^{{\gamma _k}}$ can be recalculated as

$$\begin{aligned} \dot{V}_{1,\sigma }^{{\gamma _k}} \le - {\lambda _1}e_1^2 + \frac{1}{2}{\left( {\xi _{1,\sigma }^{{\gamma _k}}} \right) ^2} + \frac{1}{2}e_2^2 + \frac{1}{2}\chi _2^2 - {k_1}{l_1}. \end{aligned}$$

*Step i*: For $2 \le i \le n - 1$, from (1) and (3), we have ${\dot{e}_i} = {\dot{x}_i} - {\dot{z}_i} = f_{i,\sigma \left( t \right) }^{\gamma \left( t \right) }\left( {{{\bar{x}}_i}} \right) + g_{i,\sigma \left( t \right) }^{\gamma \left( t \right) }\left( {{{\bar{x}}_i}} \right) {x_{i + 1}} + h_{i,\sigma \left( t \right) }^{\gamma \left( t \right) }\left( {{{\bar{x}}_i}\left( {{\tau _i}} \right) } \right) - {\dot{z}_i}$. The Lyapunov function can be chosen as follows

11 $$\begin{aligned} {V_i} = \frac{1}{2}e_i^2 + {l_i} + \frac{1}{2}\chi _i^2 + \frac{{\tilde{\theta }_i^T{{\tilde{\theta }}_i}}}{{2{\mu _i}}}, \end{aligned}$$

where ${\tilde{\theta }_i} = {\theta _i} - {\hat{\theta }_i}$ and ${l_i} = \frac{1}{2}\sum \nolimits _{j = 1}^i {{e^{ - {k_i}\left( {t - {\tau _j}} \right) }}\int _{t - {\tau _j}}^t {{e^{{k_i}s}}\beta _{i,j}^2\left( {{x_j}\left( s \right) } \right) ds} }$. Assume the *k*-th determinate subsystem is activated, denoted by $\gamma \left( t \right) = {\gamma _k}$. Then the derivative of ${V_i}$ satisfies that

12 $$\begin{aligned} \begin{aligned} \dot{V}_{i,\sigma }^{{\gamma _k}}&= {e_i}\left( {f_{i,\sigma }^{{\gamma _k}}\left( {{{\bar{x}}_i}} \right) + g_{i,\sigma }^{{\gamma _k}}\left( {{{\bar{x}}_i}} \right) {x_{i + 1}} + h_{i,\sigma }^{{\gamma _k}}\left( {{{\bar{x}}_i}\left( {{\tau _i}} \right) } \right) - {{\dot{z}}_i}} \right) + {\chi _i}{{\dot{\chi }}_i} - \frac{{\tilde{\theta }_i^T{{\dot{ \hat{\theta }} }_i}}}{{{\mu _i}}} - {k_i}{l_i}\\&\quad + \frac{1}{2}\sum \nolimits _{j = 1}^i {{e^{{k_i}{\tau _j}}}\beta _{i,j}^2\left( {{x_j}\left( t \right) } \right) - } \frac{1}{2}\sum \nolimits _{j = 1}^i {\beta _{i,j}^2\left( {{x_j}\left( {t - {\tau _j}} \right) } \right) }. \end{aligned} \end{aligned}$$

By the Assumption 2 , the inequality as follows is holds:

13 $$\begin{aligned} {e_i}h_{i,\sigma }^\gamma \left( {{{\bar{x}}_i}\left( {{\tau _i}} \right) } \right) \le \frac{1}{2}e_i^2 + \frac{1}{2}\sum \nolimits _{j = 1}^i {\beta _{i,j}^2\left( {{x_j}\left( {t - {\tau _j}} \right) } \right) }. \end{aligned}$$

Substituting (3), we get ${x_{i + 1}} = {e_{i + 1}} + {\chi _{i + 1}} + {\alpha _i}$. Then, it holds

14 $$\begin{aligned} {e_i}g_{i,\sigma }^{{\gamma _k}}\left( {{{\bar{x}}_i}} \right) {x_{i + 1}} = {e_i}g_{i,\sigma }^{{\gamma _k}}\left( {{{\bar{x}}_i}} \right) \left( {{e_{i + 1}} + {\chi _{i + 1}} + {\alpha _i}} \right) \le e_i^2{\left( {g_{i,\sigma }^{{\gamma _k}}\left( {{{\bar{x}}_i}} \right) } \right) ^2} + \frac{1}{2}e_{i + 1}^2 + \frac{1}{2}\chi _{i + 1}^2 + {e_i}g_{i,\sigma }^{{\gamma _k}}\left( {{{\bar{x}}_i}} \right) {\alpha _i}. \end{aligned}$$

By substituting (13) and (14) into (12), we have

15 $$\begin{aligned} \dot{V}_{i,\sigma }^{{\gamma _k}} \le {e_i}F_{i,\sigma }^{{\gamma _k}}\left( {{{\bar{X}}_i}} \right) + {e_i}g_{i,\sigma }^{{\gamma _k}}\left( {{{\bar{x}}_i}} \right) {\alpha _i} + {\chi _i}{{\dot{\chi }}_i} - \frac{{{{\left( {\tilde{\theta }_{i,\sigma }^{{\gamma _k}}} \right) }^T}\dot{ \hat{\theta }} _{i,\sigma }^{{\gamma _k}}}}{{\mu _{i,\sigma }^{{\gamma _k}}}} - {k_i}{l_i} + \frac{1}{2}e_i^2 + \frac{1}{2}e_{i + 1}^2 + \frac{1}{2}\chi _{i + 1}^2, \end{aligned}$$

where $F_{i,\sigma }^{{\gamma _k}}\left( {{{\bar{X}}_i}} \right) = f_{i,\sigma }^{{\gamma _k}}\left( {{{\bar{x}}_i}} \right) + {e_i}{\left( {g_{i,\sigma }^{{\gamma _k}}\left( {{{\bar{x}}_i}} \right) } \right) ^2} - {\dot{z}_i} + \frac{1}{{2{e_i}}}\sum \nolimits _{j = 1}^i {{e^{{k_i}{\tau _j}}}\beta _{i,j}^2\left( {{x_j}\left( t \right) } \right) }$. The RBF neural network is used to fit the curve of unknown function $F_{i,\sigma }^{{\gamma _k}}\left( {{{\bar{X}}_i}} \right)$. $F_{i,\sigma }^{{\gamma _k}}\left( {{{\bar{X}}_i}} \right) = {\left( {\theta _{i,\sigma }^{{\gamma _k}}} \right) ^T}\varphi _{i,\sigma }^{{\gamma _k}}\left( {{{\bar{X}}_i}} \right) + \xi _{i,\sigma }^{{\gamma _k}}\left( {{{\bar{X}}_i}} \right)$, the approximation error $\xi _{i,\sigma }^{{\gamma _k}}\left( {{{\bar{X}}_i}} \right)$ is bounded and satisfies $\xi _{i,\sigma }^{{\gamma _k}}\left( {{{\bar{X}}_i}} \right) \le {\xi _i}$. For each determinate system and stochastic system, we design the adaptive controller as

16 $$\begin{aligned} \dot{ \hat{\theta }} _{i,\sigma }^\gamma = \mu _{i,\sigma }^{{\gamma _k}}{e_i}\varphi _{i,\sigma }^\gamma \left( {{{\bar{X}}_i}} \right) . \end{aligned}$$

Invoking (15) and (16) can be equivalently rewritten as

$$\begin{aligned} \dot{V}_{i,\sigma }^{{\gamma _k}} \le {e_i}{\left( {\hat{\theta }_{i,\sigma }^{{\gamma _k}}} \right) ^T}\varphi _{i,\sigma }^\gamma \left( {{{\bar{X}}_i}} \right) + {e_i}g_{i,\sigma }^{{\gamma _k}}\left( {{{\bar{x}}_i}} \right) {\alpha _i} + {\chi _i}{{\dot{\chi }}_i} - {k_i}{l_i} + e_i^2 + \frac{1}{2}e_{i + 1}^2 + \frac{1}{2}\chi _{i + 1}^2 + \frac{1}{2}{\left( {\xi _{i,\sigma }^{{\gamma _k}}} \right) ^2}. \end{aligned}$$

*Step n*: The actual controller will be designed in the final step. By (1) and (3), the error can be computing as ${\dot{e}_n} = f_{n,\sigma \left( t \right) }^{\gamma \left( t \right) }\left( x \right) + g_{n,\sigma \left( t \right) }^{\gamma \left( t \right) }\left( x \right) u_{\sigma \left( t \right) }^{\gamma \left( t \right) } + h_{n,\sigma \left( t \right) }^{\gamma \left( t \right) }\left( {x\left( \tau \right) } \right) - {\dot{z}_n}$. Consider the Lyapunov function candidate as

17 $$\begin{aligned} {V_n} = \frac{1}{2}e_n^2 + {l_n} + \frac{1}{2}\chi _n^2 + \frac{{\tilde{\theta }_n^T{{\tilde{\theta }}_n}}}{{2{\mu _n}}}, \end{aligned}$$

where ${\tilde{\theta }_n} = {\theta _n} - {\hat{\theta }_n}$, and ${l_n} = \frac{1}{2}\sum \nolimits _{j = 1}^n {{e^{ - {k_n}\left( {t - {\tau _j}} \right) }}\int _{t - {\tau _j}}^t {{e^{{k_n}s}}\beta _{n,j}^2\left( {{x_j}\left( s \right) } \right) ds} }$. The definition of ${\theta _n}$ is given later. Assume the *k*-th determinate subsystem is activated, (18) can be derived according to (17).

18 $$\begin{aligned} \begin{aligned} \dot{V}_{n,\sigma }^{{\gamma _k}}&= {e_n}\left( {f_{n,\sigma }^{{\gamma _k}}\left( x \right) + g_{n,\sigma }^{{\gamma _k}}\left( x \right) u_\sigma ^{{\gamma _k}} + h_{n,\sigma }^{{\gamma _k}}\left( {x\left( \tau \right) } \right) - {{\dot{z}}_n}} \right) + {\chi _n}{{\dot{\chi }}_n} - \frac{{\tilde{\theta }_n^T{{\dot{ \hat{\theta }} }_n}}}{{{\mu _n}}} - {k_n}{l_n} \\&\quad + \frac{1}{2}\sum \nolimits _{j = 1}^n {{e^{{k_n}{\tau _j}}}\beta _{n,j}^2\left( {{x_j}\left( t \right) } \right) - } \frac{1}{2}\sum \nolimits _{j = 1}^n {\beta _{n,j}^2\left( {{x_j}\left( {t - {\tau _j}} \right) } \right) }. \end{aligned} \end{aligned}$$

According to Assumption 2, it can be checked that

$$\begin{aligned} {e_n}h_{n,\sigma }^\gamma \left( {x\left( \tau \right) } \right) \le \frac{1}{2}e_n^2 + \frac{1}{2}\sum \limits _{j = 1}^n {\beta _{n,j}^2\left( {{x_j}\left( {t - {\tau _j}} \right) } \right) }. \end{aligned}$$

The control input defined as $u_\sigma ^{{\gamma _k}} = {\alpha _n}$ for convenience. Following a similar process to step i, we can obtain that

19 $$\begin{aligned} \begin{aligned} \dot{V}_{n,\sigma }^{{\gamma _k}}&\le {e_n}F_{n,\sigma }^{{\gamma _k}}\left( {\bar{X}} \right) + {e_n}g_{n,\sigma }^{{\gamma _k}}\left( x \right) {\alpha _n} + \frac{1}{2}e_n^2 + {\chi _n}{{\dot{\chi }}_n} - \frac{{{{\left( {\tilde{\theta }_{n,\sigma }^{{\gamma _k}}} \right) }^T}\dot{ \hat{\theta }} _{n,\sigma }^{{\gamma _k}}}}{{\mu _{n,\sigma }^{{\gamma _k}}}} - {k_n}{l_n}\\&\le {e_n}\left( {{{\left( {\theta _{n,\sigma }^{{\gamma _k}}} \right) }^T}\varphi _{n,\sigma }^{{\gamma _k}}\left( {\bar{X}} \right) + \xi _{n,\sigma }^{{\gamma _k}}\left( {\bar{X}} \right) } \right) - \frac{{{{\left( {\tilde{\theta }_{n,\sigma }^{{\gamma _k}}} \right) }^T}\dot{\hat{\theta }} _{n,\sigma }^{{\gamma _k}}}}{{\mu _{n,\sigma }^{{\gamma _k}}}} + {e_n}g_{n,\sigma }^{{\gamma _k}}\left( x \right) {\alpha _n} + \frac{1}{2}e_n^2 + {\chi _n}{{\dot{\chi }}_n} - {k_n}{l_n}, \end{aligned} \end{aligned}$$

where $F_{n,\sigma }^{{\gamma _k}}\left( {\bar{X}} \right) = f_{n,\sigma }^{{\gamma _k}}\left( x \right) + \frac{1}{{2{e_n}}}\sum \nolimits _{j = 1}^n {{e^{{k_n}{\tau _j}}}\beta _{n,j}^2\left( {{x_j}\left( t \right) } \right) } - {\dot{z}_n}$ and the adaptive law is designed as

20 $$\begin{aligned} \dot{ \hat{\theta }}_{n,\sigma }^{{\gamma _k}} = \mu _{n,\sigma }^{{\gamma _k}}{e_n}\varphi _{n,\sigma }^{{\gamma _k}}\left( {\bar{X}} \right) . \end{aligned}$$

Now, we design the virtual control and actual control input as follows,

21 $$\begin{aligned} {\alpha _i} = \frac{{ - {\lambda _i}{e_i} - \frac{3}{2}{e_i} - {{\left( {\hat{\theta }_{i,\sigma }^{{\gamma _k}}} \right) }^T}\varphi _{i,\sigma }^\gamma \left( {{{\bar{X}}_i}} \right) }}{{g_{i,\sigma }^{{\gamma _k}}\left( {{{\bar{x}}_i}} \right) }},i = 2,3, \ldots ,n. \end{aligned}$$

For $i \in n$, consider the function $V_\sigma ^{{\gamma _k}} = \sum \nolimits _{i = 1}^n {V_{i,\sigma }^{{\gamma _k}}}$, then, with the adaptive laws, the virtual control and actual control input, we can deduce that

22 $$\begin{aligned} \begin{aligned} \dot{V}_\sigma ^{{\gamma _k}} \le&- {\lambda _1}e_1^2 + \frac{1}{2}{\left( {\xi _{1,\sigma }^{{\gamma _k}}} \right) ^2} + \frac{1}{2}e_2^2 + \frac{1}{2}\chi _2^2 - {k_1}{l_1} + \sum \nolimits _{i = 2}^n { - {\lambda _i}e_i^2 + \frac{1}{2}{{\left( {\xi _{i,\sigma }^{{\gamma _k}}} \right) }^2} - \frac{1}{2}e_i^2 + {\chi _i}{{\dot{\chi }}_i} - {k_i}{l_i} + \frac{1}{2}e_{i + 1}^2 + \frac{1}{2}\chi _{i + 1}^2} \\&- {\lambda _n}e_n^2 - \frac{1}{2}e_n^2 + \frac{1}{2}{\left( {\xi _{n,\sigma }^{{\gamma _k}}} \right) ^2} + {\chi _n}{{\dot{\chi }}_n} - {k_n}{l_n}\\ \le&\sum \nolimits _{i = 1}^n { - {\lambda _i}e_i^2 + \frac{1}{2}{{\left( {\xi _{i,\sigma }^{{\gamma _k}}} \right) }^2} - {k_i}{l_i}} + \sum \nolimits _{i = 2}^n {\frac{1}{2}\chi _i^2 + {\chi _i}{{\dot{\chi }}_i}}. \end{aligned} \end{aligned}$$

With the transformation of the virtual controller in (3), ${{\dot{\chi }}_i} = {{\dot{z}}_i} - {{\dot{\alpha }}_{i - 1}} = {{\left( {{\alpha _{i - 1}} - {z_i}} \right) } \big / {{\varsigma _i}}} - {{\dot{\alpha }}_{i - 1}} = - {{{\chi _i}} \big / {{\varsigma _i}}} - {{\dot{\alpha }}_{i - 1}}$ can be obtained. It follows that ${{\dot{\chi }}_i}{\chi _i} = - {{\chi _i^2} \big / {{\varsigma _i}}} - {{\dot{\alpha }}_{i - 1}}{\chi _i}$. There exists a non-negative continuous function $\left| {{{\dot{\chi }}_i} + {{{\chi _i}} \big / {{\varsigma _i}}}} \right| \le {\Lambda _i}\left( {{{\bar{e}}_i},{{\bar{\hat{\theta }} }_i},{{\bar{\chi }}_i},{y_d},{{\dot{y}}_d},{{\ddot{y}}_d},\rho ,\dot{\rho },\ddot{\rho }} \right)$, where ${\bar{e}_i} = \left( {{e_1},{e_2}, \ldots ,{e_i}} \right)$, ${\bar{\hat{\theta }} _i} = \left( {{{\bar{\theta }}_1},{{\bar{\theta }}_2}, \ldots ,{{\bar{\theta }}_i}} \right)$, $\bar{\chi }= \left( {{\chi _1},{\chi _2}, \ldots ,{\chi _i}} \right)$. Then we have

23 $$\begin{aligned} {\dot{\chi }_i}{\chi _i} \le - \frac{{\chi _i^2}}{{{\varsigma _i}}} + \frac{1}{2}\chi _i^2 + \frac{1}{2}\Lambda _i^2. \end{aligned}$$

Substituting (22) into (21), we get

24 $$\begin{aligned} \begin{aligned} \dot{V}_\sigma ^{{\gamma _k}}&\le \sum \nolimits _{i = 1}^n { - {\lambda _i}e_i^2 - {k_i}{l_i}} - \sum \nolimits _{i = 2}^n {\frac{1}{{{\varsigma _i}}}} \chi _i^2 + \sum \nolimits _{i = 1}^n {\frac{1}{2}{{\left( {\xi _{i,\sigma }^{{\gamma _k}}} \right) }^2}} + \sum \nolimits _{i = 2}^n {\chi _i^2 + \frac{1}{2}\Lambda _i^2} \\&\le - cV_\sigma ^{{\gamma _k}} + d, \end{aligned} \end{aligned}$$

where $$c = \mathop {\min }\limits _{i \in n} \left\{ {2{\lambda _i},2{k_i},{2 \big / {{\varsigma _i}}}} \right\}$$ and $$d = \sum \nolimits _{i = 1}^n {\frac{1}{2}{{\left( {\xi _{i,\sigma }^{{\gamma _k}}} \right) }^2}} + \sum \nolimits _{i = 2}^n {\chi _i^2 + \frac{1}{2}\Lambda _i^2}$$.

## Stability analysis

This section is devoted to analyzing and proving the stability of the entire closed-loop system. By designing the switching strategy, the controlled daul switching system achieves stability, including signal boundedness and convergence of the tracking error, and the results are summarized in Theorem 1.

**Figure 2:**
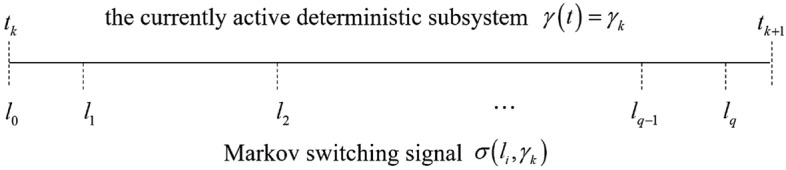
Switching timing diagram.

Let $\left\{ {{l_0},{l_1}, \ldots ,{l_q}} \right\}$ be the stochastic switching time sequence during $\left[ {{t_k},{t_{k + 1}}} \right)$. The switching timing diagram is shown in Fig. 2. Given $\Gamma \left( t \right) = {e^{c_{\sigma \left( t \right) }^{\gamma \left( t \right) }t}}V_{\sigma \left( t \right) }^{\gamma \left( t \right) }\left( {Z\left( t \right) } \right)$, obviously, it’s a piecewise differentiable along the solutions of system (1) on $\left[ {{l_i},{l_{i + 1}}} \right)$. By multiplying both sides of (24) by ${e^{ct}}$, we can get

25 $$\begin{aligned} \dot{\Gamma }\left( t \right) = c{e^{ct}}V_{\sigma \left( t \right) }^{\gamma \left( t \right) }\left( {Z\left( t \right) } \right) + c{e^{ct}}\dot{V}_{\sigma \left( t \right) }^{\gamma \left( t \right) }\left( {Z\left( t \right) } \right) \le d{e^{ct}},t \in \left[ {{\tau _i},{\tau _{i + 1}}} \right) . \end{aligned}$$

### Theorem 1

Consider the nonlinear dual switching time-delay system described by (1) with Assumptions 1–3. Controllers (10, 21), adaptive laws (9,16,20) are designed. Suppose that there exist class K functions ${\alpha _1},{\alpha _2} \in {K_\infty }$, given constants $\vartheta _i^{[j]} > 1,i \in N = \{ 1,2, \ldots ,n\} ,j \in M = \{ 1,2, \ldots ,m\}$, such that the following conditions hold:

(H1) $${\alpha _1}\left( {\left\| Z \right\| } \right) \le V_i^{\left[ j \right] }\left( Z \right) \le {\alpha _2}\left( {\left\| Z \right\| } \right) .$$

(H2) $$V_i^{[j]}\left( {e\left( t \right) } \right) \le \vartheta _i^{[j]}V_r^{[j]}\left( {e\left( t \right) } \right) .$$

Then, all signals of the closed-loop system are bounded, and the system output *y* tracks the reference signal ${y_d}$ with the desired preset performance under the following switching strategy.

(H3) $$\gamma \left( t \right) = \mathop {\arg \min }\limits _{j \in N} \left\{ {E\left[ {{V ^{\left[ j \right] }}\left( t \right) } \right] } \right\} .$$

### Proof

For $t \in \left[ {{l_q},{t_{k + 1}}} \right)$, the expectation of $V\left( {Z\left( t \right) } \right)$ is obtained by combining (25).

26 $$\begin{aligned} \begin{aligned} E\left[ {V\left( {Z\left( t \right) } \right) } \right]&= E\left[ {\Gamma \left( t \right) {e^{ - c_{\sigma \left( {{l_q}} \right) }^{{\gamma _k}}t}}} \right] = E\left[ {\left( {\Gamma _{\sigma \left( {{l_q}} \right) }^{{\gamma _k}}\left( {{l_q}} \right) + \int _{{l_q}}^t {d_{\sigma \left( {{l_q}} \right) }^{{\gamma _k}}{e^{c_{\sigma \left( {{l_q}} \right) }^{{\gamma _k}}t}}dt} } \right) {e^{ - c_{\sigma \left( {{l_q}} \right) }^{{\gamma _k}}t}}} \right] \\&= E\left[ {V_{\sigma \left( {{l_q}} \right) }^{{\gamma _k}}\left( {Z\left( {{l_q}} \right) } \right) {e^{ - c_{\sigma \left( {{l_q}} \right) }^{{\gamma _k}}\left( {t - {l_q}} \right) }}} \right] + E\left[ {\frac{{d_{\sigma \left( {{l_q}} \right) }^{{\gamma _k}}}}{{c_{\sigma \left( {{l_q}} \right) }^{{\gamma _k}}}}\left( {1 - {e^{ - c_{\sigma \left( {{l_q}} \right) }^{{\gamma _k}}\left( {t - {l_q}} \right) }}} \right) } \right] \\&\le E\left[ {\vartheta _{\sigma \left( {{l_q}} \right) }^{{\gamma _k}}V_{\sigma \left( {{l_{q - 1}}} \right) }^{{\gamma _k}}\left( {Z\left( {l_q^ - } \right) } \right) {e^{ - c_{\sigma \left( {{l_q}} \right) }^{{\gamma _k}}\left( {t - {l_q}} \right) }}} \right] + E\left[ {\frac{{d_{\sigma \left( {{l_q}} \right) }^{{\gamma _k}}}}{{c_{\sigma \left( {{l_q}} \right) }^{{\gamma _k}}}}\left( {1 - {e^{ - c_{\sigma \left( {{l_q}} \right) }^{{\gamma _k}}\left( {t - {l_q}} \right) }}} \right) } \right] \\&\le E\left[ {\vartheta _{\sigma \left( {{l_q}} \right) }^{{\gamma _k}}\Gamma \left( {{l_q}} \right) {e^{ - c_{\sigma \left( {{l_{q - 1}}} \right) }^{{\gamma _k}}{l_q}}}{e^{ - c_{\sigma \left( {{l_q}} \right) }^{{\gamma _k}}\left( {t - {l_q}} \right) }}} \right] + \frac{{d_{\sigma \left( {{l_q}} \right) }^{{\gamma _k}}}}{{c_{\sigma \left( {{l_q}} \right) }^{{\gamma _k}}}}. \end{aligned} \end{aligned}$$

In terms of (H2) in Theorem 1, integrating it over $\left[ {{t_k},{t_{k + 1}}} \right)$, by the $It\hat{o}$ formula, one gets

27 $$\begin{aligned} \begin{aligned} E\left[ {V\left( {Z\left( t \right) } \right) } \right]&\le E\left[ {\vartheta _{\sigma \left( {{l_q}} \right) }^{{\gamma _k}}\left( {\Gamma _{\sigma \left( {{l_{q - 1}}} \right) }^{{\gamma _k}}\left( {{l_{q - 1}}} \right) + \int _{{l_{q - 1}}}^{{l_q}} {d_{\sigma \left( {{l_{q - 1}}} \right) }^{{\gamma _k}}{e^{c_{\sigma \left( {{l_{q - 1}}} \right) }^{{\gamma _k}}t}}dt} } \right) {e^{ - c_{\sigma \left( {{l_{q - 1}}} \right) }^{{\gamma _k}}{l_q}}}{e^{ - c_{\sigma \left( {{l_{q - 1}}} \right) }^{{\gamma _k}}\left( {t - {l_q}} \right) }}} \right] + \frac{{d_{\sigma \left( {{l_q}} \right) }^{{\gamma _k}}}}{{c_{\sigma \left( {{l_q}} \right) }^{{\gamma _k}}}}\\&\le E\left[ {\vartheta _{\sigma \left( {{l_q}} \right) }^{{\gamma _k}}{e^{ - c_{{\sigma _q}}^{{\gamma _k}}\left( {t - {l_q}} \right) }}\left( {V_{\sigma \left( {{l_{q - 1}}} \right) }^{{\gamma _k}}\left( {Z\left( {{l_{q - 1}}} \right) } \right) {e^{ - c_{\sigma \left( {{l_{q - 1}}} \right) }^{{\gamma _k}}\left( {{l_q} - {l_{q - 1}}} \right) }} + \frac{{d_{\sigma \left( {{l_{q - 1}}} \right) }^{{\gamma _k}}}}{{c_{\sigma \left( {{l_{q - 1}}} \right) }^{{\gamma _k}}}}\left( {1 - {e^{ - c_{{\sigma _{q - 1}}}^{{\gamma _k}}\left( {{l_q} - {l_{q - 1}}} \right) }}} \right) } \right) } \right] + \frac{{d_{\sigma \left( {{l_q}} \right) }^{{\gamma _k}}}}{{c_{\sigma \left( {{l_q}} \right) }^{{\gamma _k}}}}\\&\le E\left[ {\vartheta _{\sigma \left( {{l_q}} \right) }^{{\gamma _k}}{e^{ - c_{{\sigma _q}}^{{\gamma _k}}\left( {t - {l_q}} \right) }}{e^{ - c_{{\sigma _{q - 1}}}^{{\gamma _k}}\left( {{l_q} - {l_{q - 1}}} \right) }}V_{\sigma \left( {{l_{q - 1}}} \right) }^{{\gamma _k}}\left( {Z\left( {{l_{q - 1}}} \right) } \right) } \right] + E\left[ {\vartheta _{\sigma \left( {{l_q}} \right) }^{{\gamma _k}}{e^{ - c_{{\sigma _q}}^{{\gamma _k}}\left( {t - {l_q}} \right) }}\frac{{d_{\sigma \left( {{l_{q - 1}}} \right) }^{{\gamma _k}}}}{{c_{\sigma \left( {{l_{q - 1}}} \right) }^{{\gamma _k}}}}} \right] + \frac{{d_{\sigma \left( {{l_q}} \right) }^{{\gamma _k}}}}{{c_{\sigma \left( {{l_q}} \right) }^{{\gamma _k}}}}\\&\le E\left[ {\vartheta _{\sigma \left( {{l_q}} \right) }^{{\gamma _k}}{e^{ - c_{\sigma \left( {{l_q}} \right) }^{{\gamma _k}}\left( {t - {l_q}} \right) }}{e^{ - c_{\sigma \left( {{l_{q - 1}}} \right) }^{{\gamma _k}}\left( {{l_q} - {l_{q - 1}}} \right) }}V_{\sigma \left( {{l_{q - 1}}} \right) }^{{\gamma _k}}\left( {Z\left( {{l_{q - 1}}} \right) } \right) } \right] + \vartheta _{\sigma \left( {{l_q}} \right) }^{{\gamma _k}}\frac{{d_{\sigma \left( {{l_{q - 1}}} \right) }^{{\gamma _k}}}}{{c_{\sigma \left( {{l_{q - 1}}} \right) }^{{\gamma _k}}}} + \frac{{d_{\sigma \left( {{l_q}} \right) }^{{\gamma _k}}}}{{c_{\sigma \left( {{l_q}} \right) }^{{\gamma _k}}}}\\&\vdots \\&\le E\left[ {{e^{ - c_{\sigma \left( {{l_q}} \right) }^{{\gamma _k}}\left( {t - {l_q}} \right) }}V_{\sigma \left( {{l_0}} \right) }^{{\gamma _k}}\left( {Z\left( {{l_0}} \right) } \right) \prod \limits _{i = 1}^q {\vartheta _{\sigma \left( {{l_i}} \right) }^{{\gamma _k}}{e^{ - c_{\sigma \left( {{l_{i - 1}}} \right) }^{{\gamma _k}}\left( {{l_i} - {l_{i - 1}}} \right) }}} } \right] + \sum \limits _{i = 0}^{q - 1} {\left( {\prod \limits _{j = i + 1}^q {\vartheta _{\sigma \left( {{l_j}} \right) }^{{\gamma _k}}} } \right) \frac{{d_{\sigma \left( {{l_i}} \right) }^{{\gamma _k}}}}{{c_{\sigma \left( {{l_i}} \right) }^{{\gamma _k}}}}} + \frac{{d_{\sigma \left( {{l_q}} \right) }^{{\gamma _k}}}}{{c_{\sigma \left( {{l_q}} \right) }^{{\gamma _k}}}}. \end{aligned} \end{aligned}$$

Select ${\vartheta ^{{\gamma _k}}} = \mathop {\max }\limits _{i \in N} \vartheta _{{\sigma _i}}^{{\gamma _k}}$, ${d^{{\gamma _k}}} = \mathop {\max }\limits _{i \in N} d_{{\sigma _i}}^{{\gamma _k}}$ and ${c^{{\gamma _k}}} = \mathop {\min }\limits _{i \in N} c_{{\sigma _i}}^{{\gamma _k}}$. The number of stochastic switching for the time period $\left[ {{\text{{t}}_k}\text{{, }}{\text{{t}}_{k + 1}}} \right)$ is denoted as ${N_k}$. Since ${l_0} = {t_k}$, it follows from (27) that

28 $$\begin{aligned} E\left[ {V\left( {Z\left( t \right) } \right) } \right] \le E\left[ {{e^{ - {c^{{\gamma _k}}}\left( {t - {t_k}} \right) }}{{\left( {{\vartheta ^{{\gamma _k}}}} \right) }^{{N_k}}}V_{\sigma \left( {{t_k}} \right) }^{{\gamma _k}}\left( {Z\left( {{t_k}} \right) } \right) } \right] + \frac{{{d^{{\gamma _k}}}}}{{{c^{{\gamma _k}}}}}\sum \limits _{i = 0}^{{N_k}} {{{\left( {{\vartheta ^{{\gamma _k}}}} \right) }^i}}. \end{aligned}$$

Extending the conclusion of inequality (28) to the time period $\left[ {\text{{0, t}}} \right)$ by iteration under the switching strategy in (H3), one obtains

29 $$\begin{aligned} \begin{aligned} E\left[ {V\left( {Z\left( t \right) } \right) } \right]&\le E\left[ {{e^{ - {c^{{\gamma _k}}}\left( {t - {t_k}} \right) }}{{\left( {{\vartheta ^{{\gamma _k}}}} \right) }^{{N_k}}}V_{\sigma \left( {{t_k}} \right) }^{{\gamma _k}}\left( {Z\left( {{t_k}} \right) } \right) } \right] + \frac{{{d^{{\gamma _k}}}}}{{{c^{{\gamma _k}}}}}\sum \limits _{i = 0}^{{N_k}} {{{\left( {{\vartheta ^{{\gamma _k}}}} \right) }^i}} \\&\le E\left[ {{e^{ - {c^{{\gamma _k}}}\left( {t - {t_k}} \right) }}{{\left( {{\vartheta ^{{\gamma _k}}}} \right) }^{{N_k}}}\left( {{e^{ - {c^{{\gamma _{k - 1}}}}\left( {{t_k} - {t_{k - 1}}} \right) }}{{\left( {{\vartheta ^{{\gamma _{k - 1}}}}} \right) }^{{N_{k - 1}}}}V_{\sigma \left( {{t_{k - 1}}} \right) }^{{\gamma _{k - 1}}}\left( {Z\left( {{t_{k - 1}}} \right) } \right) + \frac{{{d^{{\gamma _{k - 1}}}}}}{{{c^{{\gamma _{k - 1}}}}}}\sum \limits _{i = 0}^{{N_{k - 1}}} {{{\left( {{\vartheta ^{{\gamma _{k - 1}}}}} \right) }^i}} } \right) } \right] + \frac{{{d^{{\gamma _k}}}}}{{{c^{{\gamma _k}}}}}\sum \limits _{i = 0}^{{N_k}} {{{\left( {{\vartheta ^{{\gamma _k}}}} \right) }^i}} \\&\vdots \\&\le E\left[ {\prod \limits _{i = 0}^k {{e^{ - {c^{{\gamma _i}}}\left( {t - {t_i}} \right) }}{{\left( {{\vartheta ^{{\gamma _i}}}} \right) }^{{N_i}}}} V_{\sigma \left( {{t_0}} \right) }^{{\gamma _0}}\left( {Z\left( {{t_0}} \right) } \right) } \right] + {\left( {{\vartheta ^{{\gamma _0}}}} \right) ^{{N_0}}}{\left( {{\vartheta ^{{\gamma _1}}}} \right) ^{{N_1}}} \cdots {\left( {{\vartheta ^{{\gamma _k}}}} \right) ^{{N_k}}}\frac{{{d^{{\gamma _0}}}}}{{{c^{{\gamma _0}}}}}\sum \limits _{i = 0}^{{N_0}} {{{\left( {{\vartheta ^{{\gamma _0}}}} \right) }^i}} + \cdots \\&+ {\left( {{\vartheta ^{{\gamma _k}}}} \right) ^{{N_k}}}\frac{{{d^{{\gamma _{k - 1}}}}}}{{{c^{{\gamma _{k - 1}}}}}}\sum \limits _{i = 0}^{{N_{k - 1}}} {{{\left( {{\vartheta ^{{\gamma _{k - 1}}}}} \right) }^i}} + \frac{{{d^{{\gamma _k}}}}}{{{c^{{\gamma _k}}}}}\sum \limits _{i = 0}^{{N_k}} {{{\left( {{\vartheta ^{{\gamma _k}}}} \right) }^i}}. \end{aligned} \end{aligned}$$

Let $\vartheta = \mathop {\max }\limits _{j \in M} {\vartheta ^{\left[ j \right] }}$, $d = \mathop {\max }\limits _{j \in M} {d^{\left[ j \right] }}$ and $c = \mathop {\min }\limits _{j \in \bar{M}} {c^{\left[ j \right] }}$, one has

30 $$\begin{aligned} E\left[ {V\left( {Z\left( t \right) } \right) } \right] \le E\left[ {{e^{ - c\left( {t - {t_0}} \right) }}{\vartheta ^{N\left( {t,0} \right) }}V_{\sigma \left( {{t_0}} \right) }^{{\gamma _0}}\left( {Z\left( {{t_0}} \right) } \right) } \right] + \frac{d}{{c\left( {1 - \vartheta } \right) }}\eta , \end{aligned}$$

where $\eta = {\vartheta ^{N\left( {t,0} \right) }}\left( {1 - {\vartheta ^{{N_0} + 1}}} \right) + \cdots + {\vartheta ^{N\left( {t,{t_k}} \right) }}\left( {1 - {\vartheta ^{{N_{k - 1}} + 1}}} \right) + 1 - {\vartheta ^{{N_k} + 1}}$ and $N\left( {t,0} \right)$ represents the total number of switching in the time period $\left[ {0,t} \right)$ which is finite in switching system. The Lyapunov function $V_i^{\left[ j \right] }\left( Z \right)$ of each mode in subsystems satisfies (H1). So the following inequality (31) can be obtained.

31 $$\begin{aligned} {\alpha _1}\left( {\left\| {Z\left( t \right) } \right\| } \right) \le E\left[ {V\left( {Z\left( t \right) } \right) } \right] \le {e^{ - ct}}{\vartheta ^{N\left( {t,0} \right) }}{\alpha _2}\left( {\left\| {Z\left( 0 \right) } \right\| } \right) + \frac{d}{{c\left( {1 - \vartheta } \right) }}\eta . \end{aligned}$$

One knows that $N\left( {t,0} \right)$ are bounded in switching system. Furthermore, since $c,d,\vartheta ,\eta$ are selected positive numbers. Thus, following the similar discussion^11^, all the signals of the closed-loop system are bounded. $\square$

## Numerical simulation

In this section, an example is given to illustrate the effectiveness of the proposed approach. Consider a dual switching time-delay system with two subsystems, each with two modes, i.e., $M = 2,N = 2$.

The two modes of the first determinate subsystem are as follows:

# mode1: $$\left\{ \begin{array}{l} {{\dot{x}}_1} = {{\left( {1 - {e^{ - {x_1}}}} \right) } \big / {\left( {1 + {e^{ - {x_1}}}} \right) }} + \left( {1 + {e^{ - 1 - x_1^2}}} \right) {x_2} + 0.5x_1^2\left( {t - {\tau _1}} \right) \\ {{\dot{x}}_2} = x_1^2 - {x_2} + \left( {0.2 + 0.1\cos \left( {{x_1}} \right) } \right) u + 0.2x_2^2\left( {t - {\tau _2}} \right) \end{array} \right.$$

# mode2: $$\left\{ \begin{array}{l} {{\dot{x}}_1} = x_1^2 - 2{x_1} + \left( {0.25 + \sin \left( {{x_1}} \right) } \right) {x_2} + 0.3x_1^2\left( {t - {\tau _1}} \right) \\ {{\dot{x}}_2} = {x_1}\cos \left( {{x_2}} \right) + \sin \left( {0.5{x_2}} \right) u + {x_1}\left( {t - {\tau _1}} \right) {x_2}\left( {t - {\tau _2}} \right) \end{array} \right.$$

The two modes of the second determinate subsystem are as follows:

# mode 1: $$\left\{ \begin{array}{l} {{\dot{x}}_1} = {x_1}{e^{ - {x_1}}} + {e^{ - x_1^2}}{x_2} + {x_1}\left( {t - {\tau _1}} \right) \\ {{\dot{x}}_2} = x_2^2 - {x_1} + \left( {1 + 0.5\sin \left( {{x_2}} \right) } \right) u + 0.5{x_1}\left( {t - {\tau _1}} \right) {x_2}\left( {t - {\tau _2}} \right) \end{array} \right.$$

# mode 2: $$\left\{ \begin{array}{l} {{\dot{x}}_1} = {x_1}\sin \left( {{x_1}} \right) + \left( {0.5 + 0.3\cos \left( {{x_1}} \right) } \right) {x_2} + x_1^2\left( {t - {\tau _1}} \right) \\ {{\dot{x}}_2} = {x_1}{x_2} + \sin \left( {{x_1}} \right) u + 0.5x_2^2\left( {t - {\tau _2}} \right) \end{array} \right.$$

**Figure 3:**
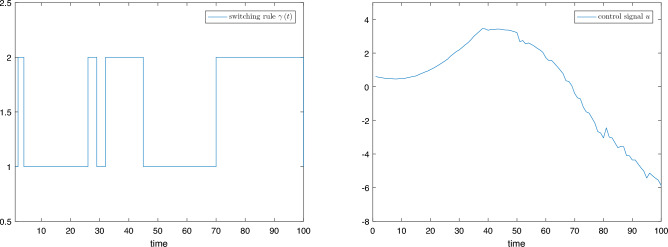
Switching rule $\gamma \left( t \right)$ and control signal $u\left( t \right)$.

**Figure 4:**
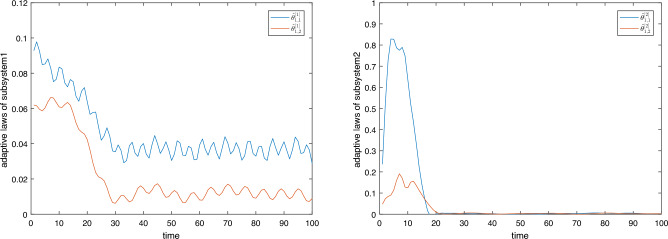
Adaptive laws of each determined subsystem.

**Figure 5:**
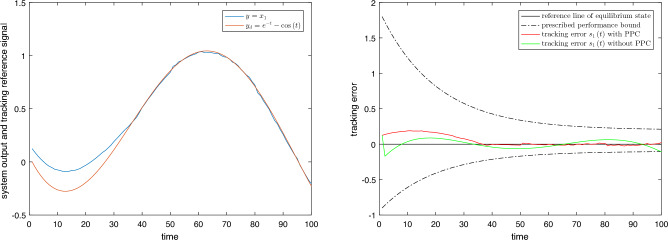
Comparison of tracking errors between PPC and without PPC.

We choose the reference signal ${y_d} = {e^{ - t}} - \cos \left( t \right)$, the time-delay ${\tau _1} = 0.2s$ and ${\tau _2} = 0.1s$, ${\beta _{1,1}}\left( {{x_1}\left( {t - {\tau _1}} \right) } \right) = x_1^2\left( {t - {\tau _1}} \right) + 1$ and ${\beta _{2,1}}\left( {{x_1}\left( {t - {\tau _1}} \right) } \right) + {\beta _{2,2}}\left( {{x_2}\left( {t - {\tau _2}} \right) } \right) = x_1^2\left( {t - {\tau _1}} \right) + x_2^2\left( {t - {\tau _1}} \right)$. Define ${\lambda _1} = {\lambda _2} = 1$ and $\mu = 1$ for all the subsystems and their modes. The parameters of prescribed performance control are selected as follows: ${\rho _0} = 1.8$, ${\rho _\infty } = 0.2$, performance function $\rho \left( t \right) = \left( {1.8 - 0.2} \right) {e^{ - t}} + 0.2$, constraints of overshoot $\underline{\delta } = 0.3,\overline{ \delta } = 0.8$.

Figure 3 shows the switching signal $\gamma \left( t \right)$ and control signal $u\left( t \right)$ designed in Theorem 1. Figure 4 shows the adaptive law $\hat{\theta }_{i,\sigma }^{{\gamma _k}}$ of each determined subsystem.

The letf subfigure of Fig. 5 shows the tracking effect of the system state output *y* on the specified reference signal ${y_d}$. It can be seen that the system output *y* can track ${y_d}$ effectively under the control of the switching signal. The right subfigure of Fig. 5 shows the tracking error. The initial value of the tracking error ${s_1}(0) = 0.125 < \rho (0) = 1.8$. It is always within the performance bound $(-\underline{\delta }\rho (t),\bar{\delta }\rho (t))$ and tends to be zero, which guarantees the dynamic and steady-state performance of the controlled system. Control accuracy comparison without PPC and with PPC is also shown in Fig. 5. Under the control of the switching signal without PPC, although the tracking error gradually becomes smaller, it oscillates near the origin. The control accuracy is not as good as PPC, and steady-state performance cannot be obtained.

## Conclusion

This paper discusses the adaptive neural network PPC problem for a class of dual switching nonlinear systems with time-delay. An adaptive NN controller based on a preset switching signal is proposed using the backstepping method, preset performance theory, and NN. The proposed scheme with preset performance can prove that all signals in the closed-loop system are bounded and the tracking error covers a small neighborhood of the origin. Simulation results verify the effectiveness of the proposed method. Our future work will focus on the PPC problem for double-switched stochastic nonlinear systems with unknown hysteresis.

## Data Availability

All data generated or analysed during this study are included in this published article.
